# Design and implementation of a generalized laboratory data model

**DOI:** 10.1186/1471-2105-8-362

**Published:** 2007-09-26

**Authors:** Michael C Wendl, Scott Smith, Craig S Pohl, David J Dooling, Asif T Chinwalla, Kevin Crouse, Todd Hepler, Shin Leong, Lynn Carmichael, Mike Nhan, Benjamin J Oberkfell, Elaine R Mardis, LaDeana W Hillier, Richard K Wilson

**Affiliations:** 1 Genome Sequencing Center, Washington University, St. Louis, MO 63108, USA

## Abstract

**Background:**

Investigators in the biological sciences continue to exploit laboratory automation methods and have dramatically increased the rates at which they can generate data. In many environments, the methods themselves also evolve in a rapid and fluid manner. These observations point to the importance of robust information management systems in the modern laboratory. Designing and implementing such systems is non-trivial and it appears that in many cases a database project ultimately proves unserviceable.

**Results:**

We describe a general modeling framework for laboratory data and its implementation as an information management system. The model utilizes several abstraction techniques, focusing especially on the concepts of inheritance and meta-data. Traditional approaches commingle event-oriented data with regular entity data in *ad hoc *ways. Instead, we define distinct regular entity and event schemas, but fully integrate these via a standardized interface. The design allows straightforward definition of a "processing pipeline" as a sequence of events, obviating the need for separate workflow management systems. A layer above the event-oriented schema integrates events into a workflow by defining "processing directives", which act as automated project managers of items in the system. Directives can be added or modified in an almost trivial fashion, i.e., without the need for schema modification or re-certification of applications. Association between regular entities and events is managed via simple "many-to-many" relationships. We describe the programming interface, as well as techniques for handling input/output, process control, and state transitions.

**Conclusion:**

The implementation described here has served as the Washington University Genome Sequencing Center's primary information system for several years. It handles all transactions underlying a throughput rate of about 9 million sequencing reactions of various kinds per month and has handily weathered a number of major pipeline reconfigurations. The basic data model can be readily adapted to other high-volume processing environments.

## Background

Over the past several decades, many of the biomedical sciences have been transformed into what might be called "high-throughput" areas of study, e.g., DNA mapping and sequencing, gene expression, and proteomics. In a number of cases, the rate at which data can now be generated has increased by several orders of magnitude. This scale-up has contributed to the rise of "big biology" projects of the type that could not have been realistically undertaken only a generation ago, e.g., the Human Genome Project [1]. Such dramatic expansions in throughput have largely been enabled by engineering innovation, e.g., hardware advancements and automation. In particular, laboratory tasks that were once performed manually are now carried out by robotic fixtures. Biologists have steadily been adopting the automated and flexible manufacturing paradigms already established in industry to increase production, as well as to reduce costs and errors.

The growing trend toward automation continues to drive the urgent need for proper software support. Here, we use the word "software" to mean those computational tools that (1) process and analyze data, (2) organize and store data and provide structured data handling capability, and (3) support the reporting, editing, arrangement, and visualization of data. This context reflects the biologist's "data → information → knowledge" paradigm [2,3]. Table 1 lists these three classes and gives an example from each that is relevant to our own specific area of interest: DNA sequencing. Each class is critical to the success of any large-scale project. The categories do not imply any procedural ordering; data that move through a processing pipeline are likely to be handled repeatedly by all three classes at various points. Moreover, certain tools may integrate parts of more than one of the classes. (This is true of some of the examples in Table 1.)

**Table 1:** Classes of Software

Class	Purpose	Example from this Class
1	process and analyze data	DNA assembler [4]
2	store, organize, and fetch data	DNA database [21]
3	arrange, edit, and view data	DNA sequence editor [5]

In the typical scenario, a research community devotes much of its attention to developing software belonging to the first group mentioned above; these applications are non-trivial in the sense that they usually involve significant algorithmic complexity. In fact, they often evolve into separate, long-term research projects in their own right. For example, although some of the earliest work in DNA sequencing software focused on the problem of fragment assembly, this area is still active [4]. Much effort is also spent on software belonging to the third group because the resulting applications directly serve end-user scientists as their primary "windows" to the data [5]. Like their counterparts in the first group, these tools normally require significant effort to build, although their research aspect is rather more limited. Developers are aided by high-level graphics languages and interfaces and designs are tailored more to end-user needs and tastes rather than specific algorithmic considerations. One can usually expect software from both groups 1 and 3 to be sophisticated and highly-refined.

In comparison, the second group is often something of a neglected middle for biological projects. This area is sometimes regarded as one of the more pedestrian hinterlands of bioniformatics, yet the underlying design issues are formidable, as we shall see below. The nucleus of group 2 is the "database", which in the present context goes by a number of colloquial names: laboratory information management system (LIMS), workflow management system, or laboratory notebook system. (Here, we will use the widely-recognized acronym "LIMS" to denote all such systems.) The basic intent is to store the entire set of laboratory processing data in a structured fashion, such that any desired subset of the data can readily be extracted or manipulated. Of course, biological LIMS are not new and there is an extensive literature covering the subject (see below).

In this paper, we report an expedient technique for modeling laboratory data and its subsequent implementation as a LIMS. This system has been developed over the last several years at our lab to support large-scale DNA mapping and sequencing, although it is not restricted to these specific uses. The model is based on a novel combination of well-known abstraction techniques and has a number of desirable features with respect to flexibility, maintainability, and process control that we describe below.

We will begin with a review of LIMS as they have been applied to biological projects thus far. This will furnish both a useful survey of and primer for LIMS design methodologies, as well as a basis of comparison for what we propose here. It is emphasized that the classifications discussed below are not strictly formal, but are meant instead to illustrate some of the possible data modeling and abstraction techniques. We then outline what we consider to be the important modeling requirements and features of a well-designed LIMS. The subsequent Results and Discussion sections introduce our model and describe its LIMS implementation and use in a high-throughput environment. Finally, project specifications are summarized in the Methods section.

### Biological LIMS: Historical summary and primer

Presumably, the LIMS concept has existed in the form of hand-written records since the earliest days of biological inquiry. The limitations of printed information in this context are obvious: systematic search and retrieval capabilities are almost completely lacking, as is the ability to cope with a large amount of data. A slightly more sophisticated approach is to use spreadsheets and text files on a filesystem. These implement what is sometimes called the *flat model *of data. Such resources continue to be employed as *ad hoc *LIMS by many small labs, where the amount and complexity of data are manageable and simple text-searching suffices. However, it is clear that the flat model remains entirely inadequate for high-throughput environments. Flat architectures are not scalable for large numbers of records, nor do they have sophisticated search capability or sufficient resolution for data tracking. Moreover, there is no ability to manage complex relationships among the data types and little flexibility to evolve as processes change. Finally, there is no straightforward method of providing transactional consistency, which is critical in preventing data corruption.

It quickly became apparent to researchers that high-throughput operations called for dedicated LIMS. Some early efforts [6] used embedded tools to organize data, e.g., the Berkeley Database Library [7], but these still did not offer a general solution. The more usual approach has been to implement a data model via a relational database management system (RDBMS). The design described by Dedhia and McCombie [8] is fairly representative. Basically, lab processes are identified, as are the materials, instruments, people, etc. that play roles in the processing pipeline. Each of these articles is then cast in a logical model as an entity type. Each type has an appropriate set of attributes and relationships between types are inferred directly from their physical manifestations. In data modeling terminology, the resulting structural description of entity types and their corresponding relationships is referred to as a *schema*.

**Figure 1:**
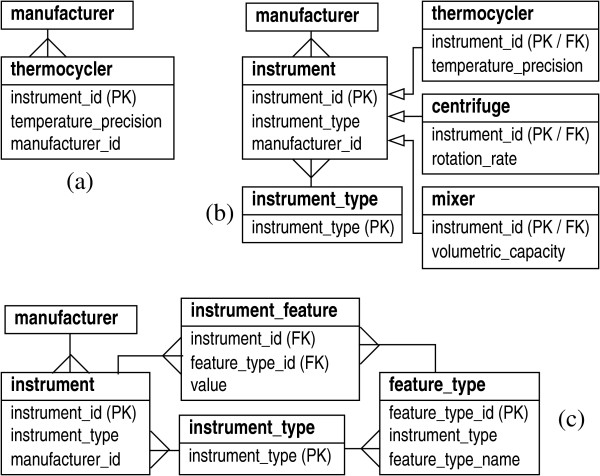
Prototypical schemas for laboratory machines: (a) direct model for an instrument, (b) using inheritance to sub-class instrument types, (c) using meta-data. Diagrams show entity type relationships and primary and foreign keys (marked 'PK' and 'FK'. respectively). The "bird's foot" symbols are a standard notation indicating that single instances from one type associate with multiple instances in the other. The "arrow" notation indicates inheritance.

A simplified portion of what such a schema might look like is shown in Fig. 1(a). Here, we focus on laboratory instruments called thermocyclers and these beget an entity type of the same name. There is a primary key (PK) called 'instrument_id' that uniquely identifies individual thermocycling instruments. Other relationships provide foreign-key (referential) constraints for additional attributes that characterize thermocyclers. For example, each one has a manufacturer, specified by 'manufacturer_id', that derives from the 'manufacturer' type. There also may be non-referentially constrained features, such as a temperature precision, e.g., ± 0.1°*C*. If more attributes are later deemed important, such as a serial number or purchase date, they can be appended to the entity type.

An entire laboratory database can readily be constructed along these lines. Once again, the main characteristic of this kind of model is that processes and entities lead directly to corresponding types and relationships. We shall refer to this class of model as the *direct model *because of the direct analogy between the physical and database realms. Direct modeling figures prominently in many of the biological LIMS reported in the literature [3][8-13]. Biological database systems routinely use direct modeling, as well [14,15].

The "direct" concept is clearly a very concrete one and this leads to certain liabilities, discussed further below. In general, a database can be improved to the degree that its design can be abstracted and several abstraction techniques are available. For example, if entity types have common subsets of attributes, the notion of *inheritance *can be applied. Common attributes are collected in a generic type, which are then referenced by keys in other, more specific types. The latter usually implement additional attributes. Fig. 1(b) shows how the basic direct model could be revised to take better advantage of inheritance. A new type called 'instrument' contains the primary key, along with attributes common to all instruments, e.g., the manufacturer. An attribute called 'instrument_type' refers to specific entity types, e.g., 'thermocycler', 'centrifuge', or 'mixer', each of which prescribes additional, instrument-specific attributes. (Here, we show the attribute 'instrument-type' as being referentially constrained by a type of the same name.) Any number of different kinds of instruments could be appended in this way. A number of LIMS designs have made conspicuous use of inheritance [16-19].

Another way to improve modeling abstraction is the use of *meta-data*. These "data that describe data" can be used to separate the structure of laboratory data from its schematic implementation. Fig. 1(c) shows the basic direct model re-cast into a form that uses meta-data. Two entity types cooperatively manage instruments in a generic fashion, for example a 'thermocycler' would now be an instantiation of an 'instrument_type' entity, while an actual thermocycler would be an instantiation of an 'instrument' type. The foreign key 'instrument_type' identifies a specific instrument as a thermocycler. Essentially, this approach recognizes that there are data that properly associate with 'instrument_type', implying that the 'thermocycler' type is superfluous at the data level. The latter is abstracted away from the schema altogether. New types of instruments would be treated by adding their meta-data descriptions to 'instrument_type'.

Although entity-specific characteristics are managed quite naturally when using inheritance, they are less obvious with meta-data. One possibility for handling attributes is shown in Fig. 1(c). A 'feature_type' type can hold instrument-specific attributes, like the temperature precision of a thermocycler. Actual feature data then reside in a bridge, in this case called 'instrument_feature', which links features to the instruments they describe. The meta-data concept is well-known, but is not often used to a degree that permits comprehensive definitions of sub-types. The emergence of dynamically typed languages for application development blends nicely with a good meta-data infrastructure, though this has apparently not been employed extensively for biological LIMS [20]. We will further discuss the uses of inheritance and meta-data below.

### LIMS requirements and schema design

At the conceptual level, good LIMS designs share several characteristics: robustness, adequate capacity to represent the complexity of the data, flexibility to evolve as materials and processes change, provision for some level of process control, and facilities to enforce, or at least support various levels of data integrity-checking. How do the approaches discussed above measure-up to these requirements?

In the first category, we count issues such as the ability to handle the required volume of data and user transactions, standardization of the interface, reasonable transaction and search times, fault-tolerance and minimal down-times, etc. Consequently, "robustness", as we use the term here, is essentially independent of schema design. Rather, it is primarily a function of the system used to implement a given data model. The relational approach has undoubtedly been the most successful in this regard, although older storage schemes such as the hierarchical architecture can still occasionally be found [21]. Relational systems have been widely developed and applied for decades and are quite mature. Commercial RDBMS, for example, have the scalability to handle enormous data sets, manage a large volume of transactions, have atomic transaction integrity, and perform with minimal down-time. Moreover, sophisticated administration tools are routinely available for backups, performance tuning, etc. Because high-throughput environments place a premium on such features, most biological LIMS have been implemented using either a commercial or a high-end, open-source RDBMS [3][8-20].

Consequently, the substantive design considerations revolve more around complexity, flexibility, process control, and data integrity. In a broad sense, these issues suggest maximal abstraction of how the data are modeled. As an illustration, let us pick a common laboratory scenario, accommodating a new type of instrument, and compare the basic direct model to one that uses meta-data. Under the simple direct model, one would create a new entity type, along with all its attributes and relevant relationships. The resulting tables and relationships would appear in the physical database and a review would be conducted to determine what user applications would need patching to maintain compatibility with the new tables. Some amount of testing and recertification of patched applications would also be expected. This is quite a labor-intensive process. In fact, Goodman et al. [19] have pointed out that if a schema changes very quickly, applications will always lag and could even be obsolete before modifications are finished. Conversely, meta-data can be strongly leveraged in this case. The new instrument type is simply an additional datum that is appended to 'instrument_type' in Fig. 1(c). The schema and physical database do not change. Moreover, the existing code infrastructure will typically handle the addition without any required changes. Consequently, code review and testing would not strictly be necessary.

These observations suggest the use of an RDBMS layed out according to design principles that provide a proper level of abstraction. The best designs will be able to resolve the data complexity, while assuring both data integrity and a high degree of flexibility to evolve as entities change. We propose a design methodology and LIMS implementation in the next section that has been formulated with these principles in mind.

## Results

A few main themes have guided much of our design work. Procedures often change much more quickly than the types of physical articles in a biological laboratory, e.g., molecules, reagents, instruments, and personnel. In fact, the latter are essentially constant in many cases. The evolution over the last 3 decades of how genomic DNA is sequenced adequately illustrates this point. Conversely, physical articles are predominantly associated with richer sets of attributes and relationships. Again, DNA in its various manifestations is a good example.

Following these observations, we have based our data model on several fundamental, somewhat hierarchical principles. First, the concepts of inheritance and meta-data are heavily exploited, often in combination with each other. Second, event tracking is separated from the tracking of physical articles, so that each can employ the most appropriate combination of abstraction techniques. Finally, the concept of an event is generalized to accommodate context-dependency. With respect to implementation, we have emphasized the object-oriented approach for the code base and a closely-coupled mapping between object classes and their counterparts in the physical database, i.e., object-relational mapping. These aspects are all described in greater detail as follows.

### Modeling of physical articles

Many of the tracking needs in a lab revolve around "physical articles". We use this term in a broad sense to mean both tangible objects and data that represent such objects, e.g. a file of DNA sequence. In general, we make conspicuous use of the notion of inheritance to model these regular entities in the lab. Physical articles can often be thought of as belonging to abstract types. For example, we discussed 'instrument' above as a type that encompasses thermocyclers, centrifuges, etc. Straightforward implementation of the model introduced in Fig. 1(b) suffices for many such cases. Additional constraints might exist, but these can sometimes be managed by trivial extension to the model. For example, one common scenario finds instances related recursively, e.g., 'person *A *reports to person *B*', which can be resolved by adding reflexive relationships.

Tangibles having richer sets of relationships can usually be captured using some combination of the abstraction techniques shown in Fig. 1. Consider that types are often related hierarchically in biological laboratories, as exemplified by DNA. Specifically, the abstract notion of 'DNA' gives rise to many concrete sub-types, e.g. raw DNA fragments, DNA ligations, subclones, sequenced DNA products, etc. Here, entities of one sub-type beget entities of another and a single instance of a DNA must fall into exactly one of these mutually-exclusive sub-types. In modeling terminology, we may say that DNA encompasses both "is-a" and "has-a" relationships. The former denotes inheritance (e.g., a subclone "is-a" DNA), while the latter represents a source-product relationship (e.g., a subclone "has-a" progenitor DNA ligation).

**Figure 2:**
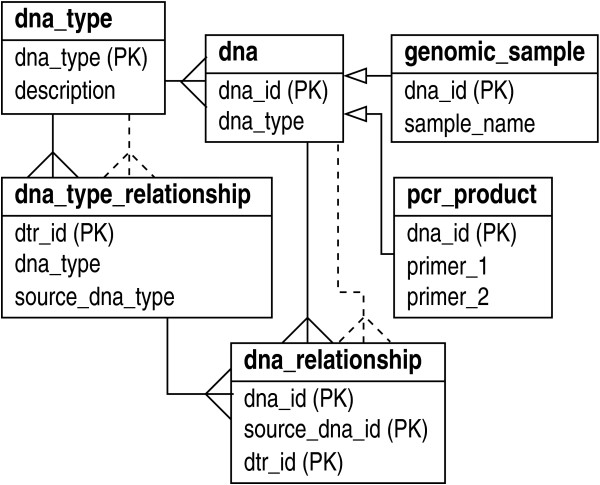
DNA as an abstract type ('dna') having hierarchical sub-types. Relationships are modeled on the basic inheritance concept with meta-data describing the hierarchy. Two sub-types are shown, 'genomic_sample' and 'pcr_product'. Each prescribes additional sub-type-specific attributes.

One design that resolves this situation is a straightforward extension of the inheritance concept that applies meta-data to describe the hierarchy (see Fig. 2). The abstract 'dna' type inherits a foreign key 'dna_type', which identifies appropriate concrete DNA types. The 'dna_type_relationship' type specifies the sub-type hierarchies, i.e., the "has-a" relationships between the different DNA types, in the form of meta-data, while 'dna_relationship' holds the instances of these relationships for actual DNA. Fig. 2 shows just two of the many possible concrete sub-types: 'genomic_sample' and 'pcr_product'. Additional sub-types would attach to the abstract 'dna' type as well, e.g., ligated products, fractioned products, and sequenced products.

One of the desirable characteristics of this design is that meta-data explicitly prescribe how the sub-types are ranked. This enables applications to infer ordering information from a query rather than resorting to internal, hard-coded logic, the significance being that it again reduces maintenance at the application level dramatically. Strictly speaking, the design enables, but does not actually enforce the hierarchical integrity of the data. That is, one could purposely connect a given DNA instance to a parent DNA instance of the wrong type. It is not practical for the RDBMS to manage this sort of constraint, but there are a number of ways to address this problem. At the most rudimentary level, one could use additional procedural code at the database level, e.g., triggers, to perform the needed validation. The more elegant solution which we prefer occurs at the Application Programming Interface (API) level, i.e., class methods (see below) validate objects before allowing data to be committed to the physical database.

The above hierarchical structure arose from the transformative nature of DNA into different types. However, other situations lead to similar hierarchical relationships that can be managed in a fashion much like that shown in Fig. 2. For example, we would expect almost all laboratories to utilize an abstract "container" type, with sub-types for trays, boxes, racks, etc. Here, the hierarchy revolves around what types of containers can reside in other types of containers, e.g., a tray resides in a box resides in a rack. This nesting specification would be encoded in the data, but enforcement would again require specific handling in the API.

### Modeling of changes and events

Recording changes and events and exactly how they occur is critical for any LIMS. We can frame this issue of "change management" with a simple analogy from human language. Consider the common characterization of database entities as nouns. Specifically, there is a table for each generic noun, with rows in each table representing proper noun instances of the generic noun and columns supplying adjectives that modify these nouns. In this context, a row is a simple atomic statement of fact, with implicit linking verbs associating the subject (the primary key) with a series of applicable adjectives. Consequently, a database can be viewed in a broader sense as a well-organized collection of facts that specifies the *state *of a system at any point in time.

Perhaps the most casual technique for event management, especially common in small-scale LIMS, is to link event data directly to the logical entities involved in the event. An additional "adjective" is often used as a descriptor. For example, 'instrument' in Fig. 1 might have a date field to log when a maintenance task last occurred. This feature does not track action directly. Rather, it defines another variable that represents the state of the entity at the time the entity is examined. This approach is a special case of the direct-modeling practice of creating complimentary database tables dedicated to tracking specific kinds of events [8,9,11].

When more detail is required, a new entity type can be defined for events occurring upon the original "noun" entity. Referring to our 'instrument' example, an event instance would be created every time maintenance is performed on a instrument. According to the language analogy, we are expanding action verbs into nouns and adjectives. For instance, the event whereby Alice repairs instrument 1000 is stored as 'instrument repair event number 1234 is done on instrument 1000 and is done by Alice'. This method has the advantage of being able to ask more complicated questions of the data. Instead of defining a set of arbitrary states and explicitly recording transition into those states, a logical function can determine if an entity is in a given state by examining the event history. This is especially useful when states are not mutually exclusive. Moreover, it allows for states to be defined *a posteriori *and re-organized as a system is re-factored.

Our design combines this basic idea with the aforementioned inheritance and meta-data strategies. We define a completely generic event type, with a distinct sub-type for each kind of event which might occur. Each sub-type is implemented as a sub-class in the API (see below), where the logic for the event is actually implemented. The base class defines the infrastructure that allows any event to associate with the required physical entities and provides an interface onto which general workflow management software can be built. Most event sub-types are almost entirely defined by their meta-data. In particular, none of the sub-types requires its own subclass-oriented table in the RDBMS and only about 20% of the sub-types actually have any code in their API subclass. More details are given in the example below.

### Process control directives

A critical consideration in the design of any data-tracking system is how specific to make event type definitions. There are two extremes, neither of which is entirely suitable. On one hand, one could allow completely generic definitions, but the significant numbers of project-specific caveats, exceptions, and re-directions would make many definitions unmanageable. Conversely, highly specific definitions would provide flexibility, but the resulting multiplication of the number of definitions raises similar management difficulties. Instead, we designed the event tracking layer to allow the attachment of "directives" to an event. Directives are instructions or parameters that govern subsequent events. They might prescribe certain parameters for a given down-stream event, veto the execution of an event entirely, or automatically prompt other events upon completion, e.g., project-specific computational analysis. A single directive can affect multiple down-stream events, and can influence a given event at multiple points in its execution. In other words, directives can function in a non-linear fashion.

The concept of the directive elegantly handles the problem of context-dependency, which routinely arises in large-scale environments. Consider the example of amplification by PCR, a process performed both in the pre-finishing stage of *de novo *genomic sequencing [1] and in the medical resequencing of patient samples [22]. While both PCRs occur in a physically identical fashion on the same instrument, each has its own specific constraints and reporting requirements. Several problems clearly loom here, including how technicians should handle an anonymously barcoded plate and how context-free logic is separated from context-specific logic.

In the API (discussed below), the directive class is abstract, requiring concrete subclasses to handle various notifications during an event's life. For example, the "project' directive has separate sub-classes for *de novo *and medical sequencing projects. Each encapsulates the appropriate constraints, extra logic, and flow control. All directives respect encapsulation, meaning that they cannot interfere with the core execution logic, except through the same parameter interface that the application uses. This feature forms a clear boundary between an event's essential parts and its peripheral ones and ensures that independent directives do not need customized integration logic.

### Overview of implementation

We have followed the standard approach for LIMS implementation, i.e., as a multi-tiered system consisting of external applications, the API, and the physical database [8]. In particular, the object-relational API translates all of the database tables into Perl software classes (packages) [23], and integrates those classes with other non-persistent and abstract classes to form the core logic layer on which the lab operates. Nearly all lab logic is in these software classes.

Although the 3-tiered layout is typical, our specific implementation of the API is appreciably different from other systems due to the explicit separation in the data model between event entities and physical articles (regular entities). Essentially, the API is divided into two parts that reflect this separation: one for the change-oriented event object layer, and the other for the regular state-oriented object layer. These layers have equal precedence from a data access perspective. However, when change occurs, the event layer is hierarchically positioned between the application and the state-object layer. Consequently, it is more accurate to speak of our system in the context of four tiers.

1. **Applications **represent interaction with users, robots, or other external agents.

2. **API Events **are used by applications to manage all change. Each action in the lab corresponds to a logged event having explicit parameters and a known software procedure responsible for the change.

3. **Regular API Entities**, usually representing physical articles, make up the state-oriented remainder of the object layer. This level is readable by applications, mutable by the event layer, and defines the state of the LIMS at any given point.

4. The **RDBMS **implements persistence.

From a programming standpoint, there are a number of interesting features native to this system. The API exploits dynamic class-loading to minimize memory usage and it maintains class meta-data to facilitate chores like dynamic generation of graphical user interfaces (GUIs). Multitable inheritance is also supported. Access to the three physical databases (see below), including distributed, multi-database transactions, is handled transparently by the software. The API also provides data type validation, field compression, and logical-to-physical field translation transparently. Software tests ensure that resolved bugs do not recur and the test battery is run automatically every hour to verify the functionality of all production code. The system also integrates the processing algorithms we use for sequence data [24]. Several other aspects of the implementation provide particular utility for high-throughput processing.

#### Bar-coding

A barcode system [25] supports detailed sample tracking. We barcode essentially everything, including small tubes and plates, agar plates, and all lab instruments, freezers, and reagent containers. Lab personnel have barcoded badges to log their ownership of events. Barcode-related information is embedded in the relational schema using a combination of the meta-data and inheritance techniques introduced in Fig. 1. We also have support for mapping external barcodes to unique identifiers in the LIMS, including two-dimensional barcodes having embedded vendor information.

#### Container management and transfer patterns

The LIMS supports event tracking down to the level of an individual well in a 96-well or 384-well plate. This provides the means to direct the specific events that should occur for single samples, as well as the ability to fully reconstruct the history of any sample. We have also defined the concept of a transfer pattern, as an instruction set that prescribes how contents are to be moved between containers. For example, a pattern might specify how components in a given sector of a 384-well plate are to be re-arrayed to a 96-well plate. Transfer patterns are mainly used to direct robotic fixtures for re-arraying and allow a simple identifier to map out complex movements of sample and reagent materials. Like barcodes, transfer patterns are embedded in the relational schema using the abstraction techniques introduced in Fig. 1.

#### Robotics integration

To the extent possible, computer systems associated with robotic fixtures in the laboratory are connected directly to the LIMS. Many of these fixtures include hardware for direct scanning of sample barcodes. For those units without this capability, technicians manually scan sample barcodes and the LIMS then utilizes the appropriate sample processing parameters to drive the robot as required. In either case, the LIMS extracts data from the robot to be integrated back into the database. One particularly notable example is our sequence machine loading application, which communicates directly with the ABI 3730xl sequencing platform (see example below). Sequence traces are extracted and automatically submitted to the GenBank Trace Archive [26] within several hours after loading.

### The life of an event: State transitions

**Figure 3:**
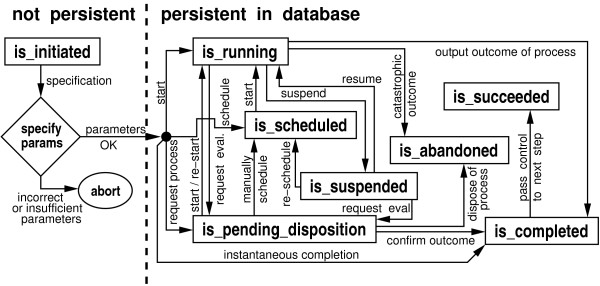
Possible state transitions for an instance of an event.

Events themselves can be thought of as state transition machines that respond to external cues and commands [27]. Fig. 3 shows a map of the state transitions that we have found useful for our particular circumstances. The novelty here is that states fall into two categories: those that are not actually recorded in the physical database ('is_initiated') and those that are recorded (all the rest). This separation provides an extra opportunity for validating information and directives at the initiation phase of a process. Specifically, at the application level, a step is created in the 'is_initiated' state, i.e., it has a reserved ID from the database and can accept parameters and processing directives as attachments. If the additions are valid, the step can proceed to subsequent, recordable states, otherwise it is aborted. The remaining states are summarized as follows.

The 'is_scheduled' state denotes that a process is waiting for available resources. In particular, it serves as a flag for a scheduling system to perform job grouping, queuing, and initiation of the actual execution of the event on a computing cluster, if required. The 'is_running' state indicates that the process instance is actually executing, for example on a compute cluster. Processes that occur instantaneously may skip this state. In 'is_suspended', a running process has been halted because of some irregularity and is waiting for error handling. This state will not appear often in highly fault-tolerant pipelines. Similarly, 'is_pending_disposition' is a flag that indicates a process is awaiting manual redirection by lab personnel. This state will not appear often in highly-automated pipelines. The 'is_abandoned' tag announces that a process has been permanently terminated before normal completion, while 'is_completed' records that a process has fulfilled all the conditions for a normal, orderly completion. The latter does not convey any information, as to the success or failure of the process. Finally, 'is_succeeded' is a sort of "super-completed" state confirming that control has successfully been transferred to a subsequent process or step.

### Aspects of implementing a process pipeline

An event is the most granular unit of change in our LIMS for which there is individual transition tracking. Broader, more complicated occurrences must be broken into suitable sequences of events, commonly referred to as "workflows" or "process pipelines". Accommodating a new task involves translating a laboratory protocol into distinct event types and defining meta-data such that these types are appropriately linked.

Linkage actually refers to both the input-output "hooks" defined in event types and the instantiated linked-list history of sequences of actual events. In particular, any new event definition is potentially connected to other, existing definitions whose output type(s) match the new event's input type(s). Various constraints may be layered over this basic requirement. For instance, if containers are involved, meta-data indicate specific container types usable in the process. The net effect is that an event is implicitly part of a pipeline if it can be executed without violating any of its internal constraints.

When a new event is initialized, the prior event from which control is being transferred is determined by finding and matching values of the event parameters. The linking of these two events is recorded in the database. This might seem superfluous, given that such connections might be inferred by queries *ad hoc*. However, querying is often not sufficient to uniquely resolve a pipeline, e.g., when samples are used across multiple projects. The consequence of such linking is that any event has access to the entire record of upstream processing from whence it came and can access this record through any of its input parameters. Furthermore, linking enables more complex constraints, such as requiring that certain precursor events have all occurred successfully before an input is accepted.

## Discussion

We have described the design and LIMS implementation of a model for laboratory data that uses a novel combination of well-known abstraction principles. Here, we provide an example of its use and discuss our experiences with it at the Genome Sequencing Center at Washington University. The LIMS has been one of the most critical factors in our success as a large-scale sequencing facility. In particular, we have a long-standing program of progressively automating laboratory workflows, especially those that are labor-intensive, and consequently, costly and error-prone. The LIMS permits us to directly monitor and control laboratory activity and to efficiently distill information for effective high-level decision-making.

### An example pipeline: Medical resequencing

**Figure 4:**
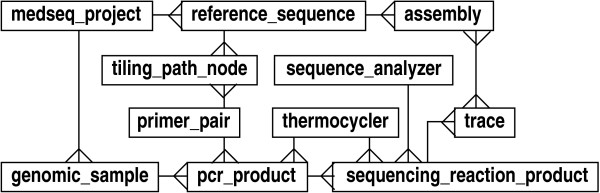
Direct modeling schema for medical sequencing projects.

The database plays a prominent role in practically every aspect of laboratory operations. Various examples of processing pipelines could be discussed, for example managing and tracking reactions for *de novo *sequencing projects. Instead, we would like to focus on a rather newer type of pipeline for medical resequencing of patient DNA. Medical resequencing is becoming increasingly important in the effort to identify causal factors of various diseases. Although the physical lab processes in this type of sequencing are similar to those for traditional genomic projects, the context is somewhat different. For example, instead of processing random clones, targeted regions of patient samples are sequenced. Moreover, the concept of an assembly is that reads are aligned to reference sequence rather than being integrated *de novo*. Fig. 4 shows an example of a direct analogy schema that could be used for a medical sequencing operation. Although the design is quite intuitive, it harbors many of the liabilities that have been discussed above. Specifically, it is quite rigid, gives little flexibility for evolving protocols, does not provide systematic event tracking, and is not readily extended to other contexts, e.g., traditional genomic sequencing.

**Figure 5:**
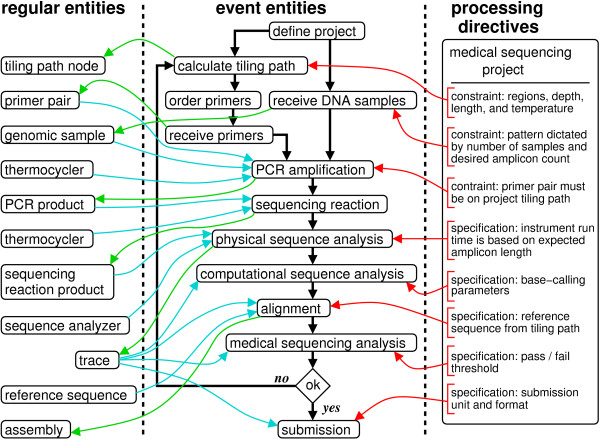
Description of a medical sequencing pipeline. Boxes represent entity instances (objects), while arrow colors represent the following: event flow (black), output from an event (green), input to an event (blue), directives governing an event (red).

The procedural layout of a typical medical sequencing operation is shown in Fig. 5. Physical articles (regular entities) and events (event entities) are represented, along with some of the specifications and constraints (directives) specific to this type of sequencing. Interactions among the various object types are complicated, demonstrating that direct designs similar to that in Fig. 4 would be inadequate. The use of directives is especially notable in this process. For example, the project object includes a list of reference sequences to target and details which portions of those reference sequences are significant. A set of primer pairs, called a tiling path, is defined such that the required regions will be covered, and this information is also part of the project. Subordinate directives are linked to the initial step in the pipeline, as well. One governs annotation of the reference sequence while another controls the tiling path algorithm. Finally, additional directives control data verification according to project-specific requirements.

The project class also has code that constrains and/or extends each of the atomic LIMS steps, based on the project object's settings. To facilitate bulk processing, project samples are queued for replication and re-arraying into 384-well plates using a transfer pattern matched to the sample count and amplification regions. Primers for the initial tiling path are ordered in parallel. Upon PCR completion, the project confirms that the correct samples are used with the correct primers and that redundant PCR is not accidentally performed. It ensures that samples which require validated primer pairs are not processed until the PCR configurations have passed a validation step in the parallel pipeline. The sample-amplicon mapping used for later analysis is also updated. This information is subsequently accessed by software on-board the ABI 3730×l sequencing analyzers to further direct how to process the samples. This includes specification of run length, which can depend on amplicon size, and a trace name, which encapsulates necessary data for use with down-stream SNP analysis tools. Although the trace analysis event performs standard processing on the traces, project directives extend processing by initiating additional analysis steps. These steps recall bases with Phred [28], compare reads to the reference sequence, determine success from the perspective of the project's goals, and record statistics.

**Figure 6:**
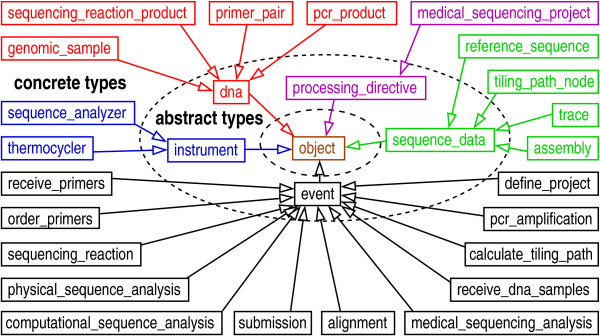
Object layout for a medical sequencing pipeline. Concrete entity types (outer-most ring) inherit from five abstract base types (middle ring). The object is in the inner-most ring. Entity types are color-coded: manifestations of DNA (red), directives (magenta), manifestations of sequence data (green), events (black), and lab instruments (blue).

**Figure 7:**
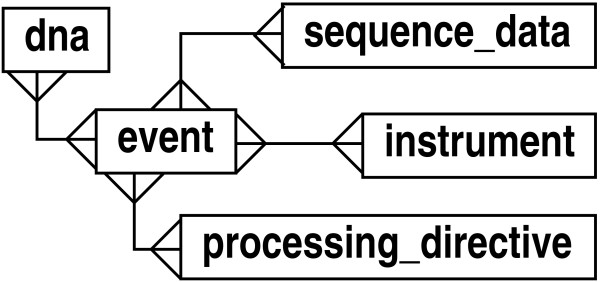
Core layout of the LIMS, showing main abstract entity types.

The object layout encompasses the totality of the logic necessary to run the pipeline (see Fig. 6). Concrete classes inherit from five highly abstracted base classes: 'dna', 'instrument', 'sequence_data', 'processing_directive', and 'event'. (Indeed, this is the general design for all of our pipelines.) In terms of actual schema implementation, the process described in Figs. 5 and 6 reduces to the simple layout shown in Fig. 7. In particular, this diagram shows the five associated abstract entity types, along with their relationships. The 'event' type provides the essential foundation from which the other types radiate via many-many relationships.

Although not shown here explicitly, each type is expanded in the database according to the various abstraction principles discussed above. For instance, 'instrument' and 'dna' are implemented according to Figs. 1(b) and 2, respectively. The 'event' type expands in a form something similar to that given by Fig. 1(c). In essence, the design of "events" provides full inheritance functionality, though it is implemented using meta-data. Again, the advantage here is that concrete event types do not require explicit table definitions in the database. This is important for maintaining flexibility in the face of rapidly evolving protocols and the consequent reconfiguring of laboratory pipelines. Notable supporting meta-data for events are input and output definitions, which can be implemented in a type slightly more general than 'feature_type' in Fig. 1(c). (For example, an attribute could be added to denote whether a definition describes an input or an output.)

### Comparison to other LIMS designs

Event-tracking is clearly one of the primary functions of any LIMS. We have described our design above and can contrast it with some of the other established systems. In many of those cases, tracking is relegated to either defining extra, *ad hoc *fields in a database table or to creating additional tables for specific kinds of tasks to hold state information [8,9,11]. This is essentially a direct approach to handling events, as illustrated by Fig. 1(a). A number of systems take this a step further by exploiting the inheritance concept. Here, a basic event specification serves as a super-class for more specific event definitions [16-18]. However, this still has the disadvantage of requiring table additions when new tasks are defined [17].

A somewhat more sophisticated approach is taken by the LabBase system [19], which provides a special layer devoted to managing materials, steps, and states. Although our software implementations are similar, LabBase appears to be significantly different from our own system at the level of the data model. Specifically, it maintains a form of stateful association with materials. Goodman et al. [19] give an example of a material-state relationship in LabBase: a 'clone' is 'ready for sequencing'. This suggests that their design does not make a strict distinction between the event itself, 'sequencing', and the state of the event, 'ready'. In actuality, LabBase is not a complete LIMS in the sense we have used the term here. Rather, it is designed to work in conjunction with a workflow management system [19], which shoulders much of the process management aspect.

Another issue that is being increasingly appreciated in biological LIMS is process control. Many of the established designs have little or no facility for using a database to directly steer events in the lab. Such configurations frequently layer a workflow management system over the database to gain some level of control [12], but this often comes in the form of altering the pipelines themselves rather than exerting a more subtle influence over a given pipeline definition.

It is well known that databases can be designed to provide certain control features [29] and some recent work has moved in this direction. In particular, the MAGIC-SPP system takes an *ad hoc *approach, designating processes as either "high-level" or "low-level" [13]. Organism-specific tasks and the processing of traces are cited as examples of these two classifications, respectively. Each division leads to its own set of dedicated tables in the database. However, it is difficult to ascertain the extent to which this design supports multiple roles, e.g., passive or non-linear control. We have also found that the ability to apply global controls over entire projects (as discussed in the medical resequencing example above) is quite advantageous. Such control would span both of the MAGIC-SPP divisions, but it is not clear how straightforward it would be to use that system in this capacity. It also does not appear that this design is readily applicable to other, non-biological workflows, e.g., processing new lab personnel or purchase orders.

### Assessment of capability and performance: Some statistics

Our LIMS has been developed over the last several years and continues to be extended. The underlying database currently has about 500 tables and functions as an On-Line Transaction Processing (OLTP) system. Many of the tables hold static data that serve as referential constraints for the data-centric tables. User applications also continue to be developed, as do the two layers of the API.

Thus far, our system has been able to keep pace with the growing throughput and complexity of our laboratory operations. A few statistics are helpful in conveying some idea of the demands that these activities place on the database. Every month, the Genome Sequencing Center performs about 9 million sequencing reactions and finishes about 50 Mb of sequence, primarily using our in-house bank of 130 ABI 3730×l sequencing machines. The OLTP database is currently about 1 terabyte in size. However, we also maintain corresponding Data Warehouse (DW) and On-Line Analytical Processing (OLAP) databases [30]. These two implementations exist for the purposes of dedicated data-mining and large-scale analyses, allowing the primary OLTP database to remain unfettered. OLTP data are copied in real-time to the DW and OLAP databases. The DW database holds only the processed results of DNA sequencing reactions: binary trace files and plain-text summary files [31], including the actual nucleotide base-calls [28]. Its schema is essentially a subset of the OLTP schema. The OLAP database holds a slightly denormalized text-only representation of these same data and is optimized for fast, large-scale analyses using a standard "star" schema [30]. In particular, reports that previously took hours to generate now run in seconds. The DW database currently contains almost 9 terabytes of data. (For comparison, it is commonly estimated that the United States Library of Congress holds the equivalent of about 20 terabytes in print form.)

We have configured our LIMS to manage event pipelines for over 20 types of entities. Most, including DNA, reagents, organisms, and purchase orders, are of a physical nature. The base-class table for DNA is the largest of these at about 1.3 billion rows. Other entities, like DNA sequencing projects, are virtual. They do not exist in a tangible sense, but nevertheless have a well-defined operational sequence. As of this writing, there are over 1800 defined processing events defined in our LIMS, of which 1300 are currently active. About 1000 of the latter are actually distinct. Aside from scheduled maintenance, database uptime has been greater than 95%.

In our experience, one of the most important features to the end-user is the ability to efficiently navigate historical information. The system we have described here supports two complimentary avenues to do this. Simple "history lists" can readily be generated starting from either a given process instance or a given material instance. For example, the predecessors of a given process can be obtained simply by traversing pointers to previous steps. Conversely, the event-history of a material can be obtained directly from that material's bridge table with the main event-tracking table. More elaborate histories can easily be constructed by combining these two approaches. Specifically, a given material's event history could be supplemented with a list of all the progenitor materials that participated in those events. Such analysis can be useful for a number of purposes, e.g., in quality assessments and tracing and troubleshooting shipment lot numbers. If so desired, one could extend this treatment *ad infinitum*, finding every progenitor material and event at every level that led to the current one.

## Conclusion

The importance of a robust, flexible LIMS for laboratory data management is only becoming more acute. Processing volumes continue to grow, processes change almost fluidly, and evolving research directions dictate increasing degrees of heterogeneity in the data. The latter point is well-illustrated by the trend toward maintaining both the traditional genomic DNA sequencing pipelines as well as medical/patient sequencing pipelines, simultaneously. These factors place enormous demands on a data-tracking system and it is only a slight exaggeration to say that an inferior LIMS can threaten a lab's very viability.

Our data model and LIMS implementation are applicable to environments that have used, or wish to use the relational approach for data management. Implementation is not limited to any particular RDBMS or programming language. In particular, we were originally prompted to choose the commercially-available Oracle RDBMS [32] because of its well-established reputation for large database projects. However, mature high-end, open-source platforms like MySQL [33] and PostgreSQL [34] now provide suitable alternatives. A generic version of our object-relational API has been released as free software [35] and efforts are now focused on extending it to support additional RDBMS platforms.

We encourage investigators to compare their existing systems to what we have reported here and to adopt any aspects that they would find to be a useful improvement.

## Methods

We have built our LIMS on the Oracle RDBMS [32], currently in version 10. The programming APIs, as well as the user applications themselves are implemented in Perl [23], currently in version 5.8.x. Graphical applications utilize the Gimp Toolkit (Gtk) library version 2.x, which is accessible through Perl, while database access is handled through Perl's DBI.pm package [36].

The OLTP database runs on four Sun Fire X4100 servers, each with 4 cores, 16 GiB of RAM, and Redhat Advance Server 4 (Nahant Update 4) as the operating system. Backups are done via NetApp Snap Vault to a NetApp R200 (disk) and by Veritas NetBackup to tape. Client applications are used mostly on commodity platforms running the Debian distribution of GNU/Linux, currently in kernel version 2.6.x.

The LIMS is managed by 14 full-time programmers and 2 database administrators (DBAs). This group represents the main non-hardware IT support for our >250-person lab. The DBAs use Oracle-proprietary tools, as well as Toad [37] and Oracletool [38] to administer the database. (The latter is an open-source application that has been modified somewhat to suit our specific needs.) Maintenance occurs daily, especially with respect to table space management. Index and table reorganization are performed as needed to maintain performance. Database queries are also monitored and timed, as needed. Statistics are collected nightly and stored in the data dictionary to facilitate query optimization. The programming environment is highly collaborative, emphasizing software engineering best practices and strict use of version control software (Subversion [39]) to manage code development.

## Availability and Requirements

Our system is available from  as a compressed Unix "tar" file, which includes schema diagrams in both PDF (Portable Document Format) and GML (Graph Modeling Language) formats, the main API framework, test cases, a demonstration program, and documentation.

**Project name: **GSC LIMS Data Model and Implementation

**Operating system: **Platform independent

**Programming language: **Perl

**Other requirements: **none

**License: **General Public License (GPL)

## Competing interests

The author(s) declares that there are no competing interests.

## Authors' contributions

All authors contributed to one or more of the primary aspects of the project: fundamental specification, model design, implementation and ongoing development, and project management and leadership. MCW, SS, CSP, and DJD drafted the manuscript. All authors read and approved the final manuscript.
